# Gp96 rich lysate as a vaccine candidate against infection with *Salmonella typhimurium*

**Published:** 2010-12

**Authors:** N Hosseini Jazani, M Karimzad, S Shahabi

**Affiliations:** 1 Center for food sciences and nutrition, Urmia University of Medical Sciences, Urmia, Iran.; 2 Department of Microbiology, Immunology and Genetics, Faculty of Medicine, Urmia University of Medical Sciences, Urmia, Iran.; 3 Center for Cellular and Molecular Research, Urmia University of Medical Sciences, Urmia, Iran.

**Keywords:** *Salmonella typhimurium*, vaccine candidate, Glycoprotein 96

## Abstract

**Background and Objectives:**

Glycoprotein 96 is the primary chaperone of the endoplasmic reticulum. Immunization with it induced potent Cytotoxic T lymphocyte responses to intracellular bacteria. *S. typhimurium* is a facultative intracellular bacterium and acquired resistance against this bacterium mainly depends on activity of Cytotoxic T cells. This study aimed to evaluate the capacity of Glycoprotein 96 rich lysate as a vaccine candidate to induce a protective immune response in mice against a lethal dose challenge with *Salmonella typhimurium*.

**Materials and Methods:**

Mice were infected with *S. typhimurium*. Then their spleens and livers were harvested and homogenized and the protein content of whole crude lysate was enriched using ammonium sulfate precipitation. SDS-polyacrylamide gel electrophoresis transfer method was used for enrichment of the protein from crude sample. Immunoblotting was conducted to detect Glycoprotein 96. Isoelectric point was achieved through the use of isoelectric focusing. PBS and whole crude lysate (from uninfected and infected mice) were injected to mice of test group, mice of control-1 group and mice of control-2 group, respectively, on days 0 and 14. Twenty-one days after the last immunization, the LD50 and bacterial loads of livers and spleens were determined****.

**Results and Conclusion:**

Immunization with Glycoprotein 96 rich lysate isolated from livers and spleens of *S. typhimurium*infected mice induced protection against infection by *S. typhimurium*. Also, the bacterial burden of livers and spleens in mice that received gp96 rich lysate significantly decreased when compared to that of mice in the control groups.

## INTRODUCTION

In humans, ingestion of various *Salmonella* serovars gives rise to infection of the small intestine followed by gastroenteritis. A small number of *Salmonella* serotypes can lead to systemic infection and enteric fever. Typhoid fever, which is caused by *Salmonella typhi*, is the prototype of such disease in humans (1).

In contrast to the severe outcome of disease in humans, *S. typhi* is avirulent in most animals, including mice. However, in mice, infection with *Salmonella typhimurium* results in enteric fever, with symptoms similar to those observed in humans after their infection with *S. typhi* (2). *S. typhimurium* infection in mice is, therefore, widely accepted as an experimental model for typhoid fever in humans (1, 3).

*S. typhimurium* is a facultative intracellular bacterium with intracellular growth and replication essential for its virulence (4). The phase of early innate immunity is followed by activation of a complex host response that suppresses the growth of bacteria in tissues. Both T lymphocytes and macrophages are involved in cell-mediated immunity to *Salmonella* infection, while antibodies also play a role (5). Although it has been shown in numerous prior studies that CD4+ T cells are of greater importance than CD8+ T cells in immunity against *S. typhimurium*. There is also evidence for participation of CD8+ T cells in immunity to *S. typhimurium* (2). Therefore, like many intracellular bacteria, acquired resistance against *S. typhimurium* depends on CD8+ T cells (2). This has created major hurdles to vaccinations using killed and antigen based vaccines (6).

Vaccination with heat shock protein (hsp)-peptide complexes could be one approach to overcome the current hurdles. The ability of heat shock proteins to: (a) chaperone peptides, including antigenic peptides; (b) interact with antigen presenting cells through a receptor; (c) stimulate antigen presenting cells to secrete inflammatory cytokines; and (d) mediate maturation of dendritic cells, permit the utilization of these proteins to develop a new generation of prophylactic and therapeutic vaccines against cancers and infectious diseases (7).

Glycoprotein 96 (Gp96), also known as glucoseregulated protein (grp94) is the primary chaperone of the endoplasmic reticulum (8). Immunization with Gp96 induced potent CTL responses to peptides of tumor antigens (9, 10), viral antigens (11–14), model antigens (15, 16), minor histocompatibility antigens (15) and intracellular bacteria (17).

We tested the capacity of Gp96 rich lysate produced from liver and spleen cells of mice infected with *S. typhimurium* as a vaccine candidate to induce a protective immune response in mice against a lethal dose challenge with *S. typhimurium*.

## MATERIALS AND METHODS

**Animal experimentation**. Six to eight-weekold male BALB/c mice were obtained from the Razi institute (Karaj, Iran). All experiments were in accordance with the Animal Care and Use Protocol of Urmia University of Medical Sciences. *S. typhimurium* PTCC (Persian Type Culture Collection) 1735 was obtained from the culture collection of The Razi Institute, Karaj, Iran. For obtaining the Gp96 rich lysate from the spleen and liver of the infected mice, twenty mice were infected with 3×106 bacterial cells via intraperitoneal injection (IP). The mean number of bacteria in all experiments was determined using the McFarland nephelometer standards (18).

**Generation of Gp96 rich lysate**. *S. typhimurium* is believed to replicate within macrophages during growth in the spleen and liver (4), so spleens and livers of the infected mice were harvested and mixed on day seven, washed twice, and homogenized with a lysis buffer (17). The lysis buffer consisted of 0.1M Tris/Hcl buffer at pH=7.8, containing 0.05% Triton X-100, 2mM EDTA and 5 µl of Protease inhibitor cocktail (Sigma). The volumes of the lysis buffer added were 5mL/mg for liver and spleen. After three freeze-thaw cycles, the whole crude lysate was centrifuged (14,000×*RPM* at 4°C for 5 min). After centrifugation, supernatant was removed and a crude sample enriched of the protein was produced using ammonium sulfate precipitation (19).

SDS-PAGE transfer method was used for enrichment of the protein from crude sample. Proteins were eluted from the gel by homogenizing as described elsewhere (20).

Following separation by SDS-PAGE, the proteins were transferred onto a PVDF membrane using a semi-dry transfer method. Only fractions containing gp96 were used for experiments. Isoelectric points were achieved through the use of isoelectric focusing (21).

The protein content of samples was determined by the Bradford method (22). Sterility testing was performed to exclude bacterial contamination of samples by culturing of the samples on TSA for 24 hours at 37°C.

**Evaluation of the immunogenicity of Gp96 rich lysate**. 144 mice were distributed into three major groups: Mice in the test group were injected subcutaneously with 50µg of Gp-96 rich lysate dissolved in PBS on days 0 and 14. Mice as control-1 group and control-2 group immunized with PBS and whole crude lysate of liver and spleen cells (50µg) of uninfected mice dissolved in PBS (Since we used the portion of lysate contained molecules with molecular weights near that of gp96 for vaccination, the lysate used as a vaccine candidate for mice of the test group was gp96 rich compared against the whole crude lysate used for mice of the control-2 group for determining whether uninfected whole cell lysate has any effects on producing immunity or not), respectively, using the same protocol. Each of the major test and control groups were divided into six subgroups.

For determining the 50% lethal doses(LD50), one week after the last immunization, 0.2-mL aliquots containing 108, 107, 106, 105, 104 or 103 cells of viable pathogenic *S.typhimurium* were prepared, and each dose of bacteria was inoculated into the 8 mice of each control and test subgroup via IP injections (3). LD50 was determined 21 days after the challenge (23).

**Determination of the bacterial loads in spleens and livers**. According to the same protocol described above, 30 BALB/c mice divided in three groups were immunized subcutaneously with Gp96 isolated from livers and spleens of infected mice (test group), PBS (control-1 group) or crude lysate from uninfected mice (control-2 group). Twenty-one days after the last immunization, the mice were infected via IP injections with 102 live *S.typhimurium*. Forty-eight hours later, mice were sacrificed, and the spleen and liver of each mouse was homogenized individually, with 10µl of appropriate dilutions [1/10 and 1/100 with Triton X-100 (0.05%)] plated on trypticase soy Agar plates. One day after culturing at 37°C, the log of colony forming units (CFUs) were determined (24).

## RESULTS

**Fig. 1:**
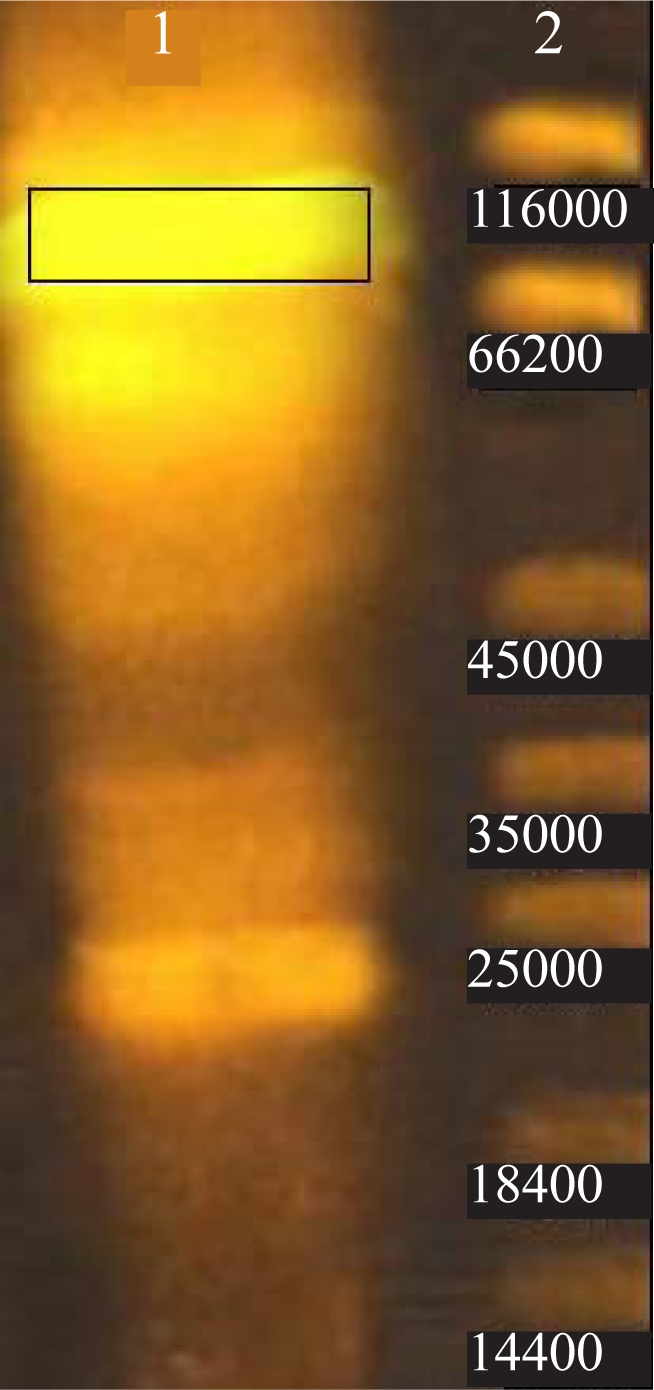
SDS-polyacrylamide gel electrophoresis (SDSPAGE) of the lysate of liver and spleen cells.1: Protein bands from crude sample. 2: protein marker with molecular mass between 14400–116000 (Fermentas).

**SDS-PAGe of the lysate of liver and spleen cells**. The results of SDS-PAGE of the lysate of liver and spleen cells showed several protein bands with different molecular weights, however bands with the molecular weight between 66,200-116,000 were cut from the gel and enriched by gel transfer method (Fig. 1).

**Fig. 2:**
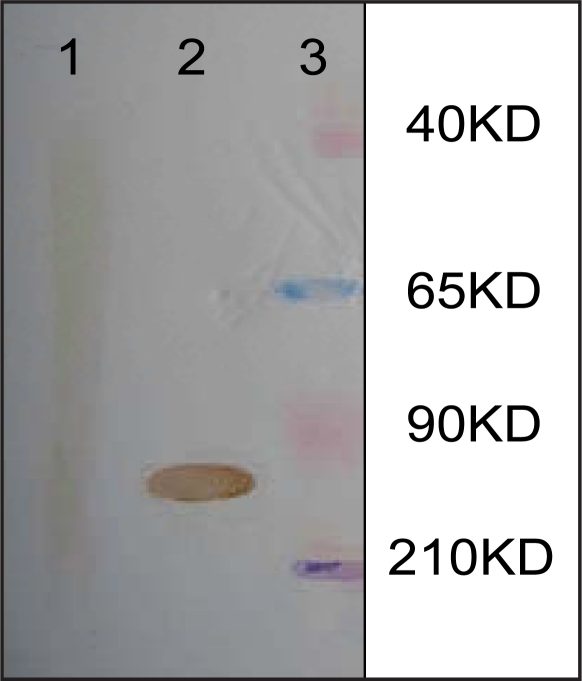
Gp96 expression of the lysate of liver and spleen cells. Western blotting using an anti-gp96 antibody indicated the existence of gp96 in the lysate of liver and spleen cells.1: negative control 2: gp96 rich lysate of spleen and liver 3: ColorBurst marker (Sigma) (210-8KDa).

**Detection of the gp96 band through the use of western blotting**. The results of western blotting using an anti-gp96 antibody indicated the existence of gp96 in the lysate of liver and spleen cells (Fig. 2).

**Fig. 3:**
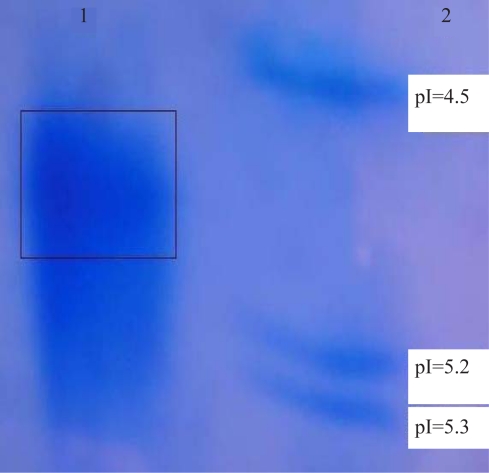
Determination of Isoelectric point of *Glycoprotein 96* rich lysate by Isoelectric focusing. 1: Gp96 rich lysate from liver and spleen cells of mice infected with *S. typhimurium*.2: IEF markers 3-10 lyophillized protein test mixture for pI determination (Serva) were used. pI 4.2: Glucose Oxidase (*A. niger*), pI 4.5: Trypsin inhibitor(soybean), pI 5.2, 5.3: Betalactoglubolin.

**Determination of Isoelectric point of Glycoprotein 96 rich lysate**. The pI of Gp96 is 4.74. So, in isoelectric focusing of the gp96 rich lysate, a heavy band between pI 4.5 and 5.2 has been shown (Fig. 3).

**Protection of mice against** ***S. typhimurium*** **challenge**. Seven days after the last immunization, survival rates of mice challenged with 103, 104, 105,106, 107 and 108 viable *S. typhimurium* inocula were analyzed (Table 1). When challenged with 105, 106,107 and 108 bacteria, the survival rates of mice in the test group were greater than those of mice in the control-1 and control-2 groups. The LD50 for the test group (2.9×107) was 1.71×103 and 1.38×103 fold greater than those of the control-1 (1.69×104) and control-2 (2.1×104) groups, respectively.

**Table 1:** The survival rate of mice immunized with PBS (control-1 group), whole crude lysate of liver and spleen cells of infected mice (control-2 group) or gp96 rich lysate (test group).

Challenge dosea^a^	Survival Rate (%)^b^		

	control-1 group	control-2 group	test group
10^3^	7/8 (87.5%)	8/8 (100%)	8/8 (100%)
10^4^	4/8 (50%)	4/8 (50%)	8/8 (100%)
10^5^	3/8 (37.5%)	2/8 (25%)	7/8 (87.5%)
10^6^	0/8 (%0)	1/8 (%12.5)	6/8 (75%)
10^7^	0/8 (%0)	0/8 (%0)	5/8 (62.5%)
10^8^	0/8 (%0)	0/8 (%0)	5/8 (62.5%)

Notes:

a Mice were challenged with the indicated dose of *S. typhimurium* (7 days after the second immunization).

b Survival rate reported 21 days after the intraperitoneal challenge with viable *S. typhimurium*.

**Fig. 4:**
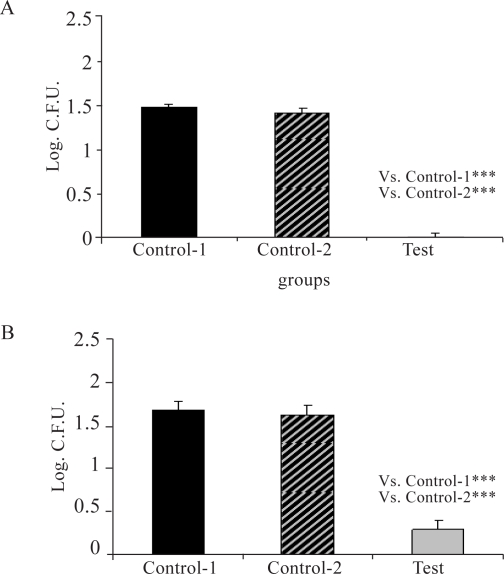
The bacterial loads in livers and spleens after challenging with *S. typhimurium. A*The log of CFUs in the culture of 10×dilutions of spleens. *B-* The log of CFUs in the culture of 10×dilutions of livers. The log CFUs indicated are the means±SEM. *** p<0.001 The mean bacterial colony counts in cultures of 0.1 homogenized livers and spleens of mice in the test group were significantly less than those of mice in the control-1 and control-2 groups (p<0.001 and p<0.001, respectively).

**Bacterial loads in the liver and spleen**. To analyze the protective effects of gp96 rich lysate against *S. typhimurium* infection, the log of the bacterial CFUs in the culture of 0.1 dilutions of homogenized livers and spleens was determined. As shown in Fig. 4-A, cultures of 0.1 homogenized spleens of mice in the test group had significantly less mean bacterial colony counts compared with those of mice in the control-1 and control-2 groups (p<0.001 and p<0.001, respectively). Additionally, the mean bacterial colony counts in cultures of 0.1 homogenized livers of mice in the test group were significantly less than those of mice in the control-1 and control-2 groups (p<0.001 and p<0.001, respectively) (Fig. 4-B). Therefore, the results indicated that the bacterial burden of livers and spleens in mice that received gp96 rich lysate as a vaccine candidate significantly decreased when compared to that of mice in the control groups.

## DISCUSSION

Heat shock proteins gp96, HSP70 and HSP90 are complexed to several cellular proteins and peptides because of their chaperone functions. Effective vaccination using *in vitro* peptide loaded heat shock proteins (HSP), tumor-derived HSP and HSP fusion proteins has been shown in viral, parasite and tumor model systems (25) ****. Vaccination with these HSPpeptide complexes induces immune responses, especially CD8+ antigen specific T cell responses against chaperoned peptide antigens. This allows for immunization with HSP-peptide complexes against tumor antigens, viruses or intracellular bacteria (12).

Our results showed that an immunization with gp96 rich lysate of liver and spleen cells of mice infected with *S. typhimurium* effectively induces protection against *S. typhimurium*. Zhang et al immunized the mice with gp96-peptide complexes extracted from different kinds of malignant tumors and the anti-tumor immunity induced by this vaccine candidate has been shown (5).

Navaratnam et al demonstrated the possibility of using gp96-peptide complexes isolated from cells expressing bovine herpes virus 1(BHV-1) proteins to induce Cytotoxic T-cells and antibody responses against BHV-1, so they showed the potential role of such preparations for vaccination against BHV-1 (26). It has also been previously reported that gp96 preparations isolated from organs of mice infected with intracellular bacteria induce cytotoxic T-lymphocyte responses and confer protection against intracellular bacteria (17). To our current knowledge, this is the first study illustrating the feasibility of vaccinating with gp96-peptide complexes against a facultative intracellular bacterial pathogen.

Zugel et al also showed the protective effects of gp96-Peptide vaccination against infection with *L monocytogenes* and *M. tuberculosis* in a mice model. They showed the feasibility of vaccinating with gp96-peptide complexes against intracellular bacteria (17), However our results confirmed these data about a facultative intracellular bacterium.

The efficacy of gp96-peptide complexes in stimulation of immune responses against *S. typhimurium* may be due to induction of cytotoxic T lymphocytes (CTLs) and T helper 1 (Th1) immune responses, by gp96-peptide complexes or gp96 by itself (17, 27). As mentioned above, CTLs play a critical role in immunity against *S. typhimurium* (2) . Also, Th1 immune responses are critical in defending against *S. typhimurium* via activation of CTLs and macrophages (2, 28) . In addition to cellular immunity, antibody response to *Salmonella* antigens participates in protection (2). On the other hand, Th1 immune responses promote shifting towards the production of antibodies, such as IgG2a, which can fight against *S. typhimurium* more efficiently (29) .

According to the results of our current study, the gp96 rich lysate of cells infected with *S. typhimurium* was an effective vaccine candidate against infection with *S. typhimurium*. Given that *S. typhimurium* infection is widely accepted as an experimental model for typhoid fever in humans (1), the gp96 rich lysate of cells infected with *S. typhi* may be a good candidate to consider as a potential vaccine for typhoid in future studies. The absence of time and expenses needed for purification of molecules and avoidance of side effects pertaining to killed or attenuated vaccines (30) are among several advantages of the vaccine candidate studied in this current research project.

To our knowledge, this study is the first of its kind to be carried out. Therefore, follow up studies are needed to confirm and extend these results.
